# Moose genomes reveal past glacial demography and the origin of modern lineages

**DOI:** 10.1186/s12864-020-07208-3

**Published:** 2020-12-02

**Authors:** Nicolas Dussex, Federica Alberti, Matti T. Heino, Remi-Andre Olsen, Tom van der Valk, Nils Ryman, Linda Laikre, Hans Ahlgren, Igor V. Askeyev, Oleg V. Askeyev, Dilyara N. Shaymuratova, Arthur O. Askeyev, Doris Döppes, Ronny Friedrich, Susanne Lindauer, Wilfried Rosendahl, Jouni Aspi, Michael Hofreiter, Kerstin Lidén, Love Dalén, David Díez-del-Molino

**Affiliations:** 1 Centre for Palaeogenetics, Svante Arrhenius väg 20C, SE-106 91 Stockholm, Sweden; 2 Department of Bioinformatics and Genetics, Swedish Museum of Natural History, Box 50007, SE-10405 Stockholm, Sweden; 3 Department of Zoology, Stockholm University, SE-10691 Stockholm, Sweden; 4 Institute for Biochemistry and Biology, University of Potsdam, Karl-Liebknecht-Str. 24-25, 14476 Potsdam, Germany; 5 Reiss-Engelhorn-Museen, Zeughaus C5, 68159 Mannheim, Germany; 6 Ecology and Genetics Research Unit, University of Oulu, P.O. Box 3000, 90014 Oulu, Finland; 7 History, Culture and Communication Studies, University of Oulu, P.O. Box 1000, 90014 Oulu, Finland; 8 Science for Life Laboratory, Department of Biochemistry and Biophysics, Stockholm University, Box 1031, SE-17121 Solna, Sweden; 9 Department of Archeology and Classical studies, Stockholm University, SE-10691 Stockholm, Sweden; 10 The Institute of Problems in Ecology and Mineral Wealth, Tatarstan Academy of Sciences, 420087 Kazan, Russia; 11 Curt-Engelhorn-Center Archaeometry, C4, 8, D-68159 Mannheim, Germany

**Keywords:** Genomics, Moose, Ancient DNA, Phylogeny, Demography

## Abstract

**Background:**

Numerous megafauna species from northern latitudes went extinct during the Pleistocene/Holocene transition as a result of climate-induced habitat changes. However, several ungulate species managed to successfully track their habitats during this period to eventually flourish and recolonise the holarctic regions. So far, the genomic impacts of these climate fluctuations on ungulates from high latitudes have been little explored. Here, we assemble a *de-novo* genome for the European moose (*Alces alces*) and analyse it together with re-sequenced nuclear genomes and ancient and modern mitogenomes from across the moose range in Eurasia and North America.

**Results:**

We found that moose demographic history was greatly influenced by glacial cycles, with demographic responses to the Pleistocene/Holocene transition similar to other temperate ungulates. Our results further support that modern moose lineages trace their origin back to populations that inhabited distinct glacial refugia during the Last Glacial Maximum (LGM). Finally, we found that present day moose in Europe and North America show low to moderate inbreeding levels resulting from post-glacial bottlenecks and founder effects, but no evidence for recent inbreeding resulting from human-induced population declines.

**Conclusions:**

Taken together, our results highlight the dynamic recent evolutionary history of the moose and provide an important resource for further genomic studies.

**Supplementary Information:**

The online version contains supplementary material available at 10.1186/s12864-020-07208-3.

## Background

Glacial and interglacial cycles had a major impact on the evolution of arctic and boreal species [1]. While many large mammals from northern latitudes went extinct toward the end of the Quaternary, several ungulates survived and subsequently recolonized the Holarctic [2]. For these species, rather than leading to extinction, glacial cycles induced range shifts and geographical isolation in refugia which resulted in inter-specific diversification or allopatric speciation [3].

Ungulates inhabiting high-latitude habitats including tundra and boreal forests are ideal models to study biogeographical processes such as vicariance and recolonisation associated with these glacial cycles. These habitats experienced important geographical shifts during glacial cycles [4, 5] and the high mobility of ungulates allowed them to quickly colonise or recolonise unglaciated areas (e.g. [6–8]). During the last glaciation, many Eurasian and North American ungulate species persisted south of the ice sheet or in southern refugia from where they recolonised areas following northward glacial retreat [6–9].

The effects of past climate changes induced contrasting responses among boreal and temperate ungulate species. For example, at the time of the Last Glacial Maximum (LGM), the range of the cold-adapted reindeer (*Rangifer tarandus*) consisted of both a continuous and large population, extending from Beringia and far into Eurasia that expanded during the Weichselian/Wisconsin glaciation some 115,000 years (115 ka) before present (BP), and of smaller refugial populations south of the ice sheets in Western Europe and in North America [6, 10]. Conversely, the population history of species from temperate climates, such as red deer (*Cervus elaphus*), fits a classical expansion-contraction model [1, 11], with contraction in isolated southern glacial refugia during cold periods followed by northward expansions during interglacials and in the Holocene [12].

Species inhabiting the taiga, such as moose (*Alces alces*), present adaptations typical of both cold-adapted and temperate species. The taiga is a boreal biome situated between the tundra and temperate habitats characterised by deciduous forests. Interestingly, variation in antler morphology of moose suggests phenotypic adaptation to open habitats, such as tundra, and to boreal forests [13]. Therefore, moose may have displayed an intermediate demographic response to past climatic changes between that of reindeer and of red deer, perhaps characterised by less severe demographic fluctuations.

Modern moose (*Alces alces*) first appear in the fossil record some 150–100 ka BP [14–16] and there is evidence that moose populations were negatively impacted by climatic changes at the end of the Pleistocene [17]. The glacial refugia moose population in Eurasia seems to have comprised two distinct genetic clades that evolved before the LGM and diversified afterwards [18]. Radiocarbon data further suggest that moose were absent from large parts of northern Europe during the LGM [19]. However, during that period, the distribution of moose extended as far south as the Italian Peninsula, the Balkans, and the Carpathians [17, 18, 20]. During the Holocene, Central and Eastern Europe were recolonised by moose from a glacial refugium in Eastern Europe and Scandinavia was recolonised from the south via a southern land bridge [20–22].

A northward shift of Eurasian boreal forests at the end of the LGM not only facilitated the recolonisation of higher latitudes but also allowed moose to colonize North America some 15–10 ka BP [19, 23–25], prior to the flooding of the Beringian land bridge some 14–11 ka BP [26]. Consistent with this hypothesis, there is no evidence of moose south of the Wisconsin glaciation ice sheets earlier than 15 ka BP in the fossil record [27]. However, the divergence between Yukon and British Columbia moose was estimated to 25–20 ka BP, and among other lineages to 17–13ka BP, suggesting that this colonisation may actually predate the end of the last glaciation [27].

While both European and North American moose populations display evidence of founder effects and bottlenecks associated with past climate changes, there is also evidence for recent anthropogenic impact on their genetic diversity [21, 28]. In Europe, the recent population history of the species was marked by a range-wide bottleneck dating back some 1800–1200 years BP and a more recent decline in moose numbers was also documented in the 18th to early 20th century [17]. Population declines during the Holocene contributed to shaping the current pattern of genetic diversity, where the Scandinavian population shows the lowest genetic diversity and highest inbreeding [17, 20]. Similarly, the species experienced a recent bottleneck in North America associated with a human-induced decline in the 1800s [27], which could potentially have exposed them to the negative effects of inbreeding.

Here, we generate the first *de-novo* reference genome for European moose and analyse it together with five previously published nuclear moose genomes to investigate patterns of genome-wide diversity and inbreeding and to test for evidence of founder effects, as well as Late Pleistocene and recent human-driven bottlenecks. We additionally generate five ancient moose mitogenomes and analyse them together with 16 previously published modern mitogenomes to study the phylogenetic relationships and divergence among Eurasian and North American moose lineages. Because of the differences in demographic history between European and North American moose as well as taxonomic uncertainties among lineages [28], this study fills an important gap in our understanding of moose evolution and population history.

## Results

### Genome assembly, genomic data and mitogenome reconstructions

The highest-quality and final *de-novo* assembly was generated with ALLPATHS_LG with a size of 2.48 Gb. It comprised 8373 scaffolds with a N50 of 1.7 Mb and had an average scaffold length of 296,974 bp. From 4104 mammalian single-copy orthologs, BUSCO showed the assembly to contain 111 (2.8%) missing, 121 (2.9%) fragmented, and 42 (1.0%) duplicated complete genes. In contrast, the assemblies generated with ABySS and SOAPdenovo had a scaffold N50 of 331.8 Kb and 316.3 Kb, respectively. Furthermore, the BUSCO analysis identified 164 (4%) and 317 (7.8%) missing, 221 (5.4%) and 256 (6.2%) fragmented, as well as 24 (0.6%) and 17 (0.4%) duplicated complete genes for the assemblies generated with ABySS and SOAPdenovo, respectively.

We identified 191 X chromosome-linked scaffolds representing 103 Mb (i.e. ~ 4.1% of the total assembly and ~ 67% of the size of the human X chromosome; Fig. S1). The average genome depth for the five nuclear genomes ranged from 12 to 20 (average = 16.6; Table S1) with the Swedish moose showing the highest depth (20-fold coverage). After filtering for missing and low-quality data, we obtained 3,204,006 high-quality SNPs segregating from the reference.

The final mitochondrial alignment was 16,693 bp long for a total of 21 mitogenomes. The newly sequenced ancient mitogenomes (*n* = 5) had a coverage ranging from 16.4 to 454.9 (Table S1).

### Population structure, divergence estimates and demographic history

**Fig. 1:**
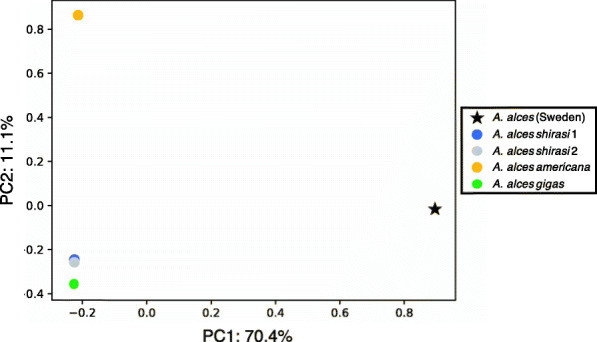
Principal Component Analysis (PCA) for five complete moose (*Alces alces*) genomes. The dataset comprised 3,204,006 high-quality SNPs

**Fig. 2:**
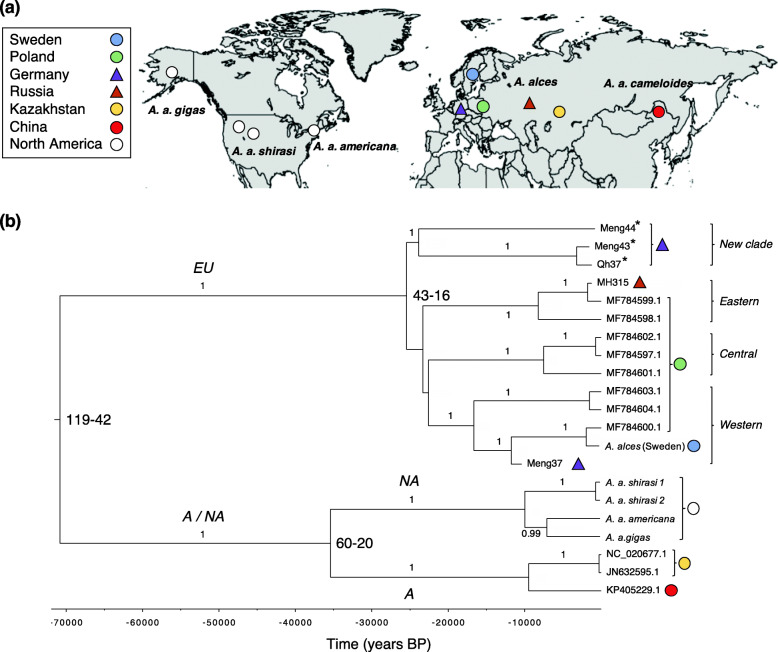
Geographical origin of 21 moose (*Alces alces*) specimens and Bayesian phylogeny based on 16,693 bp mitogenomes. **a** Circles and triangles depict modern and ancient specimens, respectively. **b** Asterisks depict samples for which dates were inferred in BEAST. Bayesian posterior probability support (over 0.9) for the branches is given. Divergence times (ka) between main lineages are given as the 95% HPD. Clades are labelled as: EU = European; A = Asian; NA = North American. Eastern, Central and Western European clades are labelled according to Świsłocka et al. [29]. The map was created using the ‘maps’ package (http://keithnewman.co.uk/r/maps-in-r.html) in R [30]

To examine the population structure of moose, we performed a Principal Component Analysis (PCA) for the five nuclear genomes and built a phylogeny for the 21 mitogenomes. The PCA based on the autosomal data indicated a clear distinction between Swedish and North American moose (Fig. 1). Also, the Eastern (*A. a. americana*) and Alaskan moose (*A. a. gigas*) showed slight genetic distinction from the other North American subspecies. Consistent with the PCA, the mitochondrial phylogeny showed a main European clade which included three previously described European sub-clades and a newly described clade composed of ancient specimens, as well as an Asian/North American clade, divided into two sub-clades (Fig. 2).

The mtDNA phylogenetic tree supported a divergence between the European and Asian/North American lineage dating back to 71 ka BP (95% Highest Posterior Density (HPD): 119–42 ka BP; Fig. 2b). The divergence between the Asian and North American lineages was estimated at 35 ka BP (95% HPD: 60–20 ka BP), and the divergence between the four European sub-clades at 25 ka BP (95% HPD: 43–16 ka BP; Fig. 2b). The estimated posterior substitution rate for the mitogenomes was 6.7 × 10^− 8^ substitutions/site/year (95% HPD: 3.53–9.84 × 10^− 8^).

**Fig. 3:**
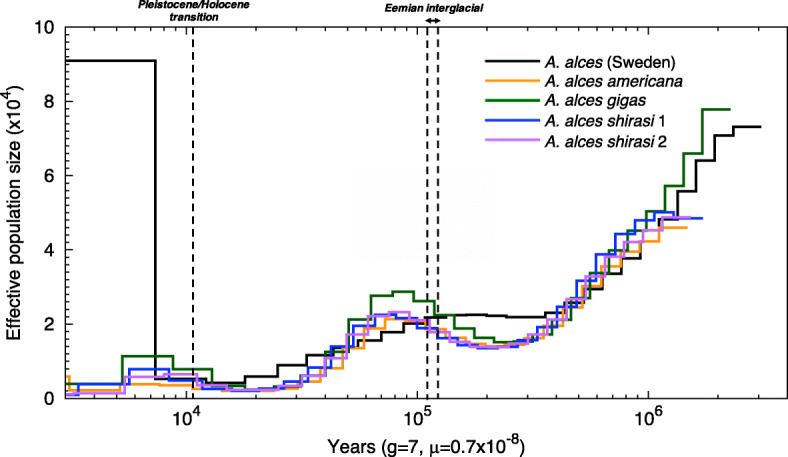
Past demography for moose (*Alces alces*) using the PSMC. Each coloured line represents a different individual. The x-axis corresponds to time before present in years on a log scale, assuming an estimated substitution rate of 0.7 × 10^− 8^ substitutions/site/generation [31] and a generation time of 7 years [32]. The y-axis corresponds to the effective population size *N*_e_

We inferred changes in effective population size (*N*_e_) of moose using the Pairwise Sequentially Markovian Coalescent (PSMC). Our results supported an increase in *N*_*e*_ for all moose populations during the Eemian interglacial, ca. 130–115 ka BP, followed by a decline during the last glacial period ca. 70 ka BP, and a small recovery at the end of the Pleistocene to a of *N*_*e*_ ~ 2000–8000 (Fig. 3, S2). While the PSMC curves indicated a shared demographic history for all moose until ca. 300 ka BP, the curve for the Swedish moose deviated from the North American ones at that time (Fig. 3).

The Bayesian Skyline Plot (BSP) based on 14 European mitogenomes indicated a constant female effective population size (*N*_ef_) over the past 30 ka BP with a median of *N*_ef_ ~ 71,400 assuming a generation time of 7 years (Fig. S3).

### Heterozygosity and inbreeding

To compare genome-wide diversity in the five moose nuclear genomes, we estimated heterozygosity and inbreeding coefficients based on the identification of runs of homozygosity (F_ROH_). The average genome-wide heterozygosity for our moose samples was 0.67 heterozygous sites per 1000 bp. The Swedish moose showed higher diversity (0.89) relative to the North American subspecies (average 0.62; Table 1).

**Table 1:** Genome-wide heterozygosity and inbreeding estimates (F_ROH_) for five moose genomes

ID	Heterozygosity* θ (95% CI)	F_ROH_ ≥ 0.5 Mb	F_ROH_ ≥ 2 Mb
*A. alces Americana (VT, USA)*	0.579 (0.559–0.582)	0.171	0.008
*A. alces gigas (AK, USA)*	0.716 (0.717–0.719)	0.082	0.006
*A. alces shirasi* 1 (WY, USA)	0.598 (0.599–0.600)	0.195	0.025
*A. alces shirasi* 2 (ID, USA)	0.586 (0.587–0.589)	0.231	0.037
*A. alces* (Sweden)	0.894 (0.896–0.897)	0.141	0.019

*Number of heterozygous sites per 1000 bp

We found low to moderate inbreeding for both Swedish and North American moose, with F_ROH_ ranging from 8 to 23% of their genome in ROH (≥0.5 Mb; Fig. S4; Table 1). When considering only long ROH (≥2 Mb), mainly arising from recent mating among close relatives, we found low inbreeding coefficients ranging between 0.6 and 3.7%, with *A. a. shirasi* showing the highest values (Fig. S4; Table 1). The maximum ROH length was 5 Mb (*A. a. shirasi* 2; Fig. S4).

## Discussion

### A reference genome for moose

Moose are the largest and heaviest extant cervid species. They play an important ecological role in the boreal and temperate circumpolar forests of Eurasia and North America, with significant impacts on boreal forest regeneration and structure [33], soil fertility [34], and predator abundance (e.g. wolf, *Canis lupus* [35, 36]). Moose are also socioculturally and economically important in many regions [37]. In Fennoscandia for example, it is one of the most intensely managed species, with up to a third of its total population killed annually [21]. Here, we generated a *de-novo* genome for the species, with a quality on par to the *de-novo* genomes of most ruminant genomes currently available (8373 scaffolds and N50 = 1.7 Mb [31]). This reference genome will be an important resource for future studies of the species’ evolutionary history as well as its suite of adaptations to the boreal environment, but it can also be a relevant tool to further refine genetic methods for monitoring and management of moose to mitigate negative impacts on boreal forests [33], maintain healthy hunting stocks [38], and for safeguarding intraspecific diversity in line with intentions of the Convention on Biological Diversity (www.cbd.int [39]).

### Moose origins and evolutionary history

Our results indicate a relatively recent divergence between European and Asian/North American moose lineages, dated to between 119 and 42 ka BP, based on modern and ancient complete mitogenomes. These dates are in agreement with an earlier divergence estimate of ca. 150–50 ka BP, based on short mtDNA data from 194 ancient radiocarbon dated samples [19], but at odds with the estimate of ca. 443 ka BP from Świsłocka et al. [29] based on complete mitogenomes. This inconsistency could be due to the fact that both our dataset and the one in Meiri et al. [19] included ancient radiocarbon dated specimens while in Świsłocka et al. [29] inferences are exclusively based on fossil-based calibration of the mutation rate based on modern data alone. Regardless, some of these divergence times have large confidence intervals, sometimes exceeding the fossil appearance of the species. The oldest *A. alces* remains are dated to 150 ka BP [15] and the latest fossils of earlier forms of *Alces* (i.e. *Cervalces latifrons)* date to ca. 186 ka BP [16, 40]. Therefore, further analysis of ancient nuclear genomes from *A. alces* and of its precursors would be needed to examine the precise timing of appearance of the European and Asian/North American moose lineages and of the origin of the species.

### Glacial refugia and modern moose lineages

Previous genetic analyses supported the existence of moose glacial refugia during the LGM in the Alps, the Caucasus, Carpathians, Balkans and northern Italy as well as in western Siberia, the Ural Mountains and Russian plains (see [18] for a review and graphical representation). However, while the taiga biome was much reduced during the LGM in Eurasia, pollen and macrofossil data indicate that the taiga was characterised by a wide yet discontinuous geographical range in the form of isolated patches further east, in southern Ukraine and the Urals, Western Siberia, northern Mongolia and in eastern Siberia over most of the last glacial period [41, 42], potentially indicating the presence of several fragmented refugial moose populations throughout Eurasia. A higher genetic diversity in East Asia, estimated from ancient mtDNA, is also consistent with a LGM refugium in Siberia that could have extended to northern China [19]. Our dataset, combining ancient and modern mitogenomes, allowed us to identify one main European moose lineage composed of up to four clades diverging just before the LGM, ca. 25 ka BP, including three clades described in Świsłocka et al. [29] and one new, previously unknown clade. Sample MH315 from Tatarstan (1227 years BP) is part of the Eastern clade together with two samples from Poland. The Central clade is represented by three samples from eastern Poland. The modern Swedish moose belongs to the Western European clade. Even though limited to one sample, this supports the hypothesis that contemporary Scandinavian moose originate from a refugial population in western/central Europe via a southern colonization that happened after the last glaciation [43]. In fact, our oldest ancient moose sample from Germany, Meng37 (10 ka BP), is also part of this Western clade, indicating that this clade was present in central Europe at the onset of the Holocene. Finally, we describe a new European clade composed solely of three ancient moose samples from Germany dated to 1557–810 years BP (Meng43, Meng44, Qh37). While we caution about the accuracy of these dates, based on a limited number of radiocarbon dated specimens, it is intriguing that this clade is absent among modern moose, thereby suggesting that it disappeared during the Holocene. Such entire clade disappearance has previously been shown in brown bears (*Ursus arctos* [44]). Conversely, this clade indicates that there could have been more glacial refugia for moose in central Europe than previously identified.

Based on mtDNA data, we estimate a divergence between the Asian and North American moose lineages dating back to ca. 35 ka BP. This estimate predates the colonisation of the North American continent by moose, inferred from short mtDNA sequences and estimated at ca. 15 ka BP [28, 45] but it coincides with the divergence of human lineages in Asia prior to the colonisation of North America [46–48]. This suggests the existence of genetic structure in the Asian lineage prior to the North American colonisation via Beringia, where the first *Alces* fossils are dated to 15 ka BP [23]. Similar to Meiri et al. [19], our mitochondrial phylogenetic tree suggests that all modern North American moose derive from a single migration event. However, we find some genetic differentiation among North American subspecies in the PCA, with Eastern moose (*A. a. americana*) and Alaskan (*A. a. gigas*) moose being distinct from the other North American samples which form a tight cluster. We hypothesize that this differentiation could be a consequence of the founder effects during the colonisation of North America. Sequencing of additional ancient and modern genomes from eastern Siberia, Beringia and North America will be essential to solve these questions.

### Past demography

Our results indicate that the past 150 ka of moose history was significantly influenced by glacial cycles. Overall, changes in *N*_e_ inferred with the PSMC, indicate that moose primarily responded to glacial cycles in the same way as temperate species such as red deer (*C. elaphus*), with expansions during interglacials and contractions in cryptic and isolated refugia during glacials [12]. For example, we found evidence for a demographic expansion possibly coinciding with the onset of the Eemian interglacial, which was followed by a decline ca. 70 ka BP. Interestingly, the lack of moose fossil remains from Europe during the LGM is consistent with either a demographic decline [19] or a shift in geographical distribution. Thus, it is possible that moose demographic history largely reflects historical shifts in boreal forests and the taiga during cold periods, including the last glaciation [19]. Following the decline, moose populations experienced a slight demographic recovery, some 15–10 ka BP at the Pleistocene/Holocene transition. Importantly, Meiri et al.’s [19] observation of a temporal gap in radiocarbon dates across Asia, but only two base pairs difference between two pre- and post-LGM moose from northern Siberia supports the hypothesis of habitat tracking in response to ice sheet movements. The moose population may thus have tracked its habitat as it shifted south during the LGM and then expanded north after the ice receded, instead of populations in the north going extinct and subsequently being replaced as climate conditions improved after the LGM. However, additional genomic data from diverse Eurasian moose populations would be required to further test this hypothesis.

There were striking differences between the demographic trajectories of Swedish and North American moose. While the Swedish moose experienced a gradual decline from the Eemian up to the Pleistocene/Holocene transition, the North American moose genomes showed signatures of increase in *N*_e_ during the Eemian and declined later, ca. 50 ka BP. The different trajectories between the two moose could be artefactual. However, since moose had not colonised North America at the time [28, 49], this difference could be an indication of different population histories between the European and Asian populations. For example, European moose seem to have experienced a stronger range contraction than the Asian moose during the last glaciation [19]. We also found evidence for population expansions in both European and North American moose at the end of the LGM, ca. 15 ka BP, which likely reflects the northward advances as the species tracked its habitat, similar to previous advances during warming periods, some 59 ka BP and 14 ka BP, as shown by Hundertmark et al. [28].

We caution that PSMC is known to lack power in estimating *N*_e_ in very recent times (i.e. < 10 ka BP) due to fewer coalescent events [50]. We bypassed this limitation by reconstructing female demographic changes using Bayesian inference based on the mitogenome data in European moose. We found a constant *N*_ef_ over the past 30 ka BP, which is consistent with previous analyses of modern European mitochondrial data [18, 20]. Therefore, our results support a scenario where European moose experienced a spatial rather than a demographic expansion, with only limited founder effects, as they shifted their range northwards at the end of the LGM. Finally, while the confidence intervals for the *N*_ef_ estimates were wide, we note a disconnect between a mitochondrial *N*_ef_ of ~ 71,000 and a nuclear *N*_e_ of 2000-8000. This difference could be due to variance in male reproductive success in a highly polygynous species such as moose, with a mating system characterized by intrasexual competition, and where successful males maintain harems [51, 52].

### Moose genomic diversity

We found that the majority of ROH were short (< 2 Mb) in all moose genomes, which indicates that most of the observed inbreeding is due to background relatedness [53, 54], potentially resulting from glacial or post-glacial bottleneck events [17, 20, 28, 43]. For example, the three American samples showed the highest background inbreeding and lowest diversity, consistent with a single or several founder effects when colonizing North America at the end of the last glaciation [27]. Interestingly, the Alaskan moose, *A. a. gigas,* had the lowest inbreeding and highest diversity of all North American moose samples. This could indicate that moose colonised Alaska from a large refugial population, possibly in Beringia and that they subsequently experienced serial founder effects in their colonisation of the rest of North America. In fact, both the Swedish and Alaskan moose displayed the highest levels of heterozygosity compared to the other moose specimens, suggesting the existence of large refugial populations in Europe and Beringia during the LGM [17, 20, 55].

We only detected low levels of inbreeding arising from long ROH (≥2 Mb), commonly caused by recent mating with relatives during bottlenecks [53, 54]. This result is at odds with previous studies which identified limited mitochondrial and nuclear diversity in moose populations from Europe [21, 28] and North America [27, 56]. Moreover, evidence for a severe reduction in effective population size to less than 3% of their former size (down to *N*_e_ ~ 400), lasting for hundreds of generations and possibly dating back as early as the fifteenth century, has been previously reported in Swedish moose populations [21]. Similarly, substantial reductions in *N*_*e*_ in moose populations from Lapland and northern Finland have been associated with 18th century bottlenecks [57]. Interestingly, Norwegian moose populations do not seem to have experienced such population reductions [58, 59]. It is thus possible that recent admixture with some of these populations may have prevented an increase in inbreeding in the Swedish population. Recent moose sightings in southeastern Germany and the expansion of populations from Poland further suggest that the species has potential for long range dispersal [60]. Altogether, our results suggest that the recent demographic history of European moose may be more complicated than that of a single bottleneck scenario.

While inbreeding was generally low, it is however worth noting that the two Rocky Mountain moose (*A. a. shirasi*) specimens had the largest inbreeding coefficients and the longest ROH (~ 4–5 Mb). This is consistent with these southernmost North American populations being founded from relatively few individuals after the declines of the late 1800s and with low gene flow from northern populations until the middle of the 20th century [27].

## Conclusion

In this study, we present a reference genome for the European moose, which will serve as an important resource for monitoring as well as for future studies on the evolutionary history and population genomics of the species. Through analysis of several moose genomes, we provide a glimpse into the demography and population history of the species in Europe and North America. Our results indicate that current moose lineages trace back their origin to several refugial populations during the LGM. Throughout their history, moose have experienced similar population demographic fluctuations as temperate ungulates (e.g. red deer, *C. elaphus* [12]). However, their overall spatial response to the Pleistocene/Holocene transition are more consistent with a range shift, reflecting historical shifts in the taiga.

## Methods

### Sampling and data collection

We used stored muscle tissue from a young female moose from Sweden (Province of Gävleborg, Central Sweden; approximate RT90 coordinates 1570800/6855900) to generate a *de-novo* assembly (see below; Table S1). We also obtained bone samples from five ancient moose specimens from Russia (*n* = 1) and Germany (*n* = 4; Table S1). Radiocarbon dating of one German sample (Meng37) was performed at the Curt-Engelhorn-Centre for Archaeometry gGmbH (CEZA) laboratory at the Reiss-Engelhorn-Museen in Mannheim (sample identifiers starting with “MAMS”) using a MICADAS AMS system [61]. Conventional ^14^C ages were calibrated to calendar ages with the IntCal13 data set [62] and the SwissCal software (L. Wacker, ETH Zurich). Radiocarbon dating for the Russian sample (MH315) was performed at the Oxford Radiocarbon Accelerator Unit (sample identifiers starting with “OxA”). The AMS-date was turned into calendar years using the IntCal13 calibration curve in Oxcal v4.2 [63].

We then supplemented our newly generated data with published nuclear and mitochondrial genome data for four North American moose (ENA project number: PRJNA325061; Table S1) and 11 mitogenomes for Eurasian and North American moose (Table S1). We therefore used two different datasets: a genomic dataset comprising the nuclear genomes from five modern moose samples (one newly sequenced), and a mitogenomic dataset composed of 21 samples in total - 16 modern and five ancient ones (six newly sequenced; Fig. 2a, Table S1).

### DNA extraction and sequencing

In order to generate a *de-novo* assembly, we extracted total DNA from muscle tissue from the female moose using a Thermo Scientific KingFisher Duo magnetic particle processor (ThermoFisher Scientific) with the KingFisher Cell and Tissue DNA Kit. We then prepared two paired-end libraries with 180 bp inserts using a TruSeq DNA kit (Illumina, CA, USA) according to the manufacturer’s specification, but automated on an Agilent NGS workstation (Agilent, CA, USA). We also constructed two mate-pair libraries according to the Nextera gel-plus protocol (Illumina, CA, USA) using a Blue Pippin (Sage Science Inc., MA, USA) for size selecting target fragments: one 2.5–4.5 kb mate-pair library (MPS) and one 5–7 kb mate-pair library (MPL) using the 0.75% 1–10 kb Gel Cassette with Marker S1. All libraries were indexed to enable de-multiplexing after sequencing. These libraries were sequenced on an Illumina HiSeqX v.2.5 instrument (HiSeq Control Software 3.3.76/RTA 2.7.6) with a 2 × 150 bp setup, where the 180 bp library was sequenced on 1.5 lanes. The mate-pair libraries were multiplexed in equimolar ratios and sequenced on 0.5 lanes (in total) at Sci*Life*Lab (Stockholm, Sweden). BCL to FASTQ conversion was performed using BCL2FASTQ v1.8.3 from the CASAVA software suite.

DNA from the ancient sample from Russia (MH315; Table S1) was extracted using the silica-column protocol described in Yang et al. [64] and modified in Gamba et al. [65]. A USER treated genomic library [66] was then built following Meyer & Kircher [67] and subjected to mitogenome capture as described in Maricic et al. [68], using deer-specific baits and as described in Heino et al. [69]. The resulting library was purified, quantified on a 2100 Bioanalyzer (Agilent) and sequenced on a HiSeq lane with paired-end 2 × 126 bp setup.

DNA was extracted from the petrous bones of four additional ancient samples from Germany (Meng37, Meng43, Meng44, Qh37; Table S1) following Dabney et al. [70]. Single stranded libraries were prepared according to Gansauge et al. [71] and pre-treated with 0.5 μl of USER enzyme (New England Biolabs, Ipswich, MA, USA) for 15 min at 37 °C to remove uracil residues resulting from post-mortem damage (modified from Meyer et al. [72]). The resulting libraries were then amplified and indexed using two double-unique p5-p7 tailed primers to generate dual-indexed library molecules. The number of PCR cycles was determined in advance from qPCR results, as described in Gansauge & Meyer [73]. Amplified libraries were then pooled in equimolar amounts according to their concentration and length distribution determined using Qubit 2.0 and 2200 TapeStation (Agilent Technologies), respectively. Finally, libraries were sequenced on an Illumina NovaSeq6000 S4 platform using 2 × 100 bp paired-end setup at Sci*Life*Lab (Stockholm, Sweden).

### De-novo reference genome assembly

The raw sequencing reads were processed using Trimmomatic v0.32 [74] to remove low quality sequences and adaptor read-through. Trimmed reads were assembled using three different methods: ALLPATHS-LG r.52488 [75] with the option “HAPLOIDIFY = True”, ABySS v1.3.5 [76] and SOAPdenovo 2.04-r240 [77]. For assembly evaluation we used QUAST v4.5.4 [78] and BUSCO v3.0.2 [79] with the “mammalia_odb9” dataset. The assembly showing the highest contiguity and best BUSCO scores (Genbank project number PRJNA668262; Table S1) was used for mapping of resequenced data and downstream analyses.

We used the genomic dataset to identify the X chromosome-linked scaffolds of the moose reference genome using their relative genomic coverage as in Malde et al. [80]. Briefly, we estimated which scaffolds larger than 10 kb displayed ca. half of the coverage than the genomic average in the three male moose samples but ca. the average in the two female samples (Fig. S1).

### Bioinformatic procedures

In order to generate complete nuclear moose genomes, we mapped paired-end short read data for the newly generated (ENA Project number: PRJNA40679; Table S1) and the four downloaded moose samples (ENA Project number: PRJNA325061; Table S1) to our *de-novo* moose assembly. To avoid biases, we processed both the newly generated and downloaded data using the same bioinformatic pipeline. Briefly, forward and reverse reads were trimmed to remove Illumina adapter sequences using Trimmomatic v0.32 with default settings [74] and then mapped to the reference genome using BWA mem v0.7.13 [81]. We then used SAMtools v1.8 [82] for coordinate sorting, indexing, and removing duplicates from the alignments. Next, we re-aligned reads around indels using GATK IndelRealigner v3.4.0 [83]. For all downstream analyses, we only retained reads with mapping quality ≥30. We estimated the depth of coverage for the five genomes using Qualimap v2.2.1 [84].

Next, we used bcftools v1.8 [82, 85] to call variants for each moose genome. We retained SNPs with a minimum depth of coverage of 1/3 of the average coverage and maximum depth of 10 times the average coverage of each individual genome. We also retained SNPs with base quality ≥30 and excluded those within 5 bp of indels. For all downstream analyses, we excluded all scaffolds (*n* = 191) identified as linked to X chromosomes. We also hard masked all repeat regions across the genome using BEDtools v2.27.1 [86]. Finally, we merged all five individuals into one single vcf file keeping only those positions genotyped in all samples using PLINK v1.9 [87].

For the mitochondrial dataset, we mapped the raw sequencing data from the ancient moose specimens sequenced here (Table S1) to the mitogenome of an Eurasian moose (Genbank accession number: MF784604.1). We used BWA aln v0.7.8 [81] with settings adjusted for ancient DNA studies as described in Pečnerová et al. [88]. After removing PCR duplicates with a python script which takes into account both the start and end of the alignments (github.com/pontussk/samremovedup), we generated consensus sequences from the BAM files in ANGSD v0.921 [89] using the majority rule (*−doFasta 2)* and a minimum depth threshold of 3. To avoid biases, in the four ancient moose samples subjected to partial USER treatment (Meng37, Meng43, Meng44, Qh37), which leaves post-mortem damage at the fragments termini, we trimmed 1 bp from both sides of each sequence before mapping.

The mitogenomes from the five modern moose for which shotgun sequencing data was available (newly sequenced and downloaded) were generated as above, but with the following modifications: mapping was performed with BWA mem, PCR duplicates were excluded using SAMtools, and the minimum depth threshold was set to 100. The final mitochondrial alignment of modern and ancient newly sequenced samples and downloaded sequences (*n* = 21) was generated in MAFFT v7.407 [90].

### Population structure and phylogenetic analyses

We first used the R package SNPRelate [91] to perform a Principal Component Analysis (PCA) based on the genetic covariance matrix estimated from the genotypes using our filtered SNP genomic dataset.

Secondly, we used BEAUti/BEAST v1.8.4 [92] to build a phylogeny for the 21 mitogenomes. In order to estimate the molecular age of three undated samples (Meng43, Meng44 and Qh37), we created a ‘taxa’ partition of these undated samples. We set the median value of the dates for the two radiocarbon dated samples as calibrated tip dates (Table S1) and used the ‘sampling with individual priors’ for the undated samples partition (Meng43, Meng44, Qh37). We determined the best fitting evolutionary model for the mitogenome dataset with jModelTest v2.1.9 [93] and selected a model of HKY + G using the Bayesian Inference Criterion as previously used by DeCesare et al. [27]. We used a constant size model with a strict molecular clock and set the clock rate to a normal distribution with a mean value at 9 × 10^− 9^ substitutions/site/year based on Zurano et al. [94] and a standard deviation of 0.01. The ages of the three ancient samples without radiocarbon dates were determined using a uniform prior ranging from 0 to 100 ka BP. We then ran all models using BEAST v1.8.4 for 50 million generations with sampling every 1000 generations. To ensure that convergence had occurred, we visualized all output log files in Tracer v1.7.1 [95] and then combined all trees in a single tree with LogCombiner v1.8.4. We used TreeAnnotator v1.10.4 [96] to remove the first 10% of runs as burn-in from the tree files. Finally, we visualised and built the phylogenies in Figtree v1.4.4 (github.com/rambaut/figtree).

### Demography and divergence estimates

To reconstruct past changes in effective population size (*N*_e_) of moose, we used the Pairwise Sequentially Markovian Coalescent (PSMC v0.6.5 [50]) model and applied it to the five moose nuclear genomes. This model identifies historical recombination events across a diploid genome and infers the time to the most recent common ancestor (TMRCA) between independent segments of the genome. Assuming that pairwise sequence divergence is proportional to the time of the coalescent event, regions of low heterozygosity correspond to recent coalescent events while regions of high heterozygosity correspond to more ancient coalescent events. Because the rate of coalescence is inversely proportional to *N*_e_, it can then be used to estimate temporal changes in *N*_e_.

After excluding all sites in the scaffolds (*n* = 191) identified as linked to the X chromosome, we generated consensus sequences for all autosomes of the five modern genomes using the SAMtools mpileup [82] command and the vcf2fq command from vcfutils.pl. We then excluded sites with base quality and mapping quality below 30, and depth below 1/3 of the average coverage, which was estimated for each genome independently. Since PSMC’s model is highly sensitive to false heterozygous sites, for this analysis we used a more strict threshold of maximum depth excluding positions with more than two times the average coverage estimated for each genome. We set N (the number of iterations) = 25, t (Tmax) = 15 and p (atomic time interval) = 64 (4 + 25*2 + 4 + 6, for each of which parameters are estimated with 28 free interval parameters) for the inference of TMRCA between each chromosome from each individual genome. We scaled population parameters assuming a generation time of 7 years [32] and a substitution rate of 0.7 × 10^− 8^ substitutions/site/generation, inferred from a rate of 1 × 10^− 9^ substitutions/site/year from Chen et al. [31].

Given that PSMC results are less accurate at times more recent than 10 ka BP [50], we also estimated fluctuations in female effective population size (*N*_ef_) using the mitogenomes from 14 European moose using BEAUti/BEAST v1.8.4 [92]. We used the same parameters as described above for the phylogenetic tree but used a Bayesian Skyline Plot (BSP) model. We then visualised all output log files with Tracer v1.7.1 [95] to ensure that convergence had occurred and to generate the BSP.

### Heterozygosity and inbreeding

In order to estimate the individual genomic diversity we used mlRho v2.7 [97]. mlRho allows to estimate the population mutation rate (θ), which approximates the per-site expected heterozygosity under the infinite sites model [97]. We excluded bases with quality below 30, reads with mapping quality below 30 and positions with root-mean-squared mapping quality below 30 from modern bam files and excluded all scaffolds identified as linked to X chromosomes. We also filtered out sites with depth lower than 1/3 of and higher than 10 times the average coverage. This allowed us to avoid biases associated with erroneous mapping to the reference genome and with false heterozygous sites caused by variable coverage across the genome which can result from structural variation.

We then quantified individual inbreeding coefficients by estimating the number and lengths of runs of homozygosity (ROH). ROH are long genomic tracts without heterozygous sites that can be used to inform about past and recent inbreeding levels [53, 54]. We first converted the filtered multi-individual vcf file comprising the five nuclear genomes into a ped file and identified all ROH in autosomal scaffolds. We then used PLINK v1.9 [87] to identify ROH with the following settings: a sliding window size of 1000 SNPs (*homozyg-window-snp 1000*); a maximum of 3 heterozygous sites per window (*homozyg-window-het 3*); a minimum of 5% of overlapping windows that must be called homozygous to define any given SNP as ‘in a homozygous segment’ (*homozyg-window-threshold 0.05*); a minimum of 10 SNP per window (*homozyg-snp 10*); a minimum of 500 kb coverage (*homozyg-kb 500*); a minimum SNP density of one SNP per 50 kb (*homozyg-density 50*); a maximum distance between two neighbouring SNPs of 1000 kb (*homozyg-gap 1000*); a maximum of 999 heterozygous sites within ROH of 999 (*homozyg-het 999*). Finally, we calculated individual inbreeding coefficients (F_ROH_) as the overall proportion of their genome contained in ROH by summing the size of all ROH per individual divided by the total genome size (i.e. autosomes only). We considered two ROH size cut-offs at ≥0.5 Mb and ≥ 2 Mb, representing background relatedness from matings between distant relatives and recent inbreeding events, respectively [53, 54].

## Supplementary Information

**Additional file 1.**

**Additional file 2:** **Figure S1.** Identification of scaffolds linked to chromosome X using sequencing coverage and scaffold length. Green dots correspond to scaffolds assigned as linked to chromosome X. The red lines indicate the median coverage for all scaffolds. The blue lines represent half the median coverage which corresponds to the coverage of scaffolds linked to chromosome X. **Figure S2.** Past demography for moose (*Alces alces*) using PSMC. Thin lines represent 100 bootstrap runs. The x-axis corresponds to time before present in years on a log scale, assuming an estimated substitution rate of 0.7 × 10^− 8^ substitutions/site/generation [30] and a generation time of 7 years [31]. The y-axis corresponds to the effective population size *N*_e_. **Figure S3.** Past demography for moose (*Alces alces*) using a Bayesian Skyline Plot (BSP). Demographic reconstruction was inferred in BEAST using 14 European 16,693 bp mitogenomes. Timing of events was estimated assuming a mean rate of 9 × 10^− 9^ substitutions/site/year based on Zurano et al. [93] and a standard deviation of 0.01. The x axis is in calendar years before present and y axis represents changes in effective population size (shown as the product of *N*_ef_ and generation time T). The black line corresponds to the median estimate and the blue lines show the 95% highest posterior density intervals. **Figure S4.** Distribution of runs of homozygosity (ROH) in moose (*Alces alces*). ROH ≥500 kb are shown.

## Data Availability

*de-novo* reference genome for European Moose (*A. alces*): Genbank accession project number PRJNA668262 (Table S1). Raw paired-end trimmed fastq data for one complete nuclear genome and five mitogenomes: ENA (https://www.ebi.ac.uk/ena) project accession number PRJEB40679 (Table S1).

## Abbreviations

LGM: Last glacial maximum
BP: Before present
PCA: Principal component analysis
HPD: Highest posterior density
PSMC: Pairwise sequentially markovian coalescent
TMRCA: Time to the most recent common ancestor
BSP: Bayesian skyline plot
*N*_e_: Effective population size
*N*_ef_: Female effective population size
ROH: Runs of Homozygosity
F_ROH_: Inbreeding coefficients based on the identification of Runs of Homozygosity
