# Supplementary material for: Moose genomes reveal past glacial demography and the origin of modern lineages
Source: BMC Genomics. 2020 Dec 2;21:854. doi: 10.1186/s12864-020-07208-3 (PMC7709250; doi:10.1186/s12864-020-07208-3)
Supplement: Supplementary file 2 — Additional file 2: Figure S1. Identification of scaffolds linked to chromosome X using sequencing coverage and scaffold length. Green dots correspond to scaffolds assigned as linked to chromosome X. The red lines indicate the median coverage for all scaffolds. The blue lines represent half the median coverage which corresponds to the coverage of scaffolds linked to chromosome X. Figure S2. Past demography for moose (Alces alces) using PSMC. Thin lines represent 100 bootstrap runs. The x-axis corresponds to time before present in years on a log scale, assuming an estimated substitution rate of 0.7 × 10− 8 substitutions/site/generation [30] and a generation time of 7 years [31]. The y-axis corresponds to the effective population size Ne. Figure S3. Past demography for moose (Alces alces) using a Bayesian Skyline Plot (BSP). Demographic reconstruction was inferred in BEAST using 14 European 16,693 bp mitogenomes. Timing of events was estimated assuming a mean rate of 9 × 10− 9 substitutions/site/year based on Zurano et al. [93] and a standard deviation of 0.01. The x axis is in calendar years before present and y axis represents changes in effective population size (shown as the product of Nef and generation time T). The black line corresponds to the median estimate and the blue lines show the 95% highest posterior density intervals. Figure S4. Distribution of runs of homozygosity (ROH) in moose (Alces alces). ROH ≥500 kb are shown. [file 12864_2020_7208_MOESM2_ESM.docx]

**Supplementary material**


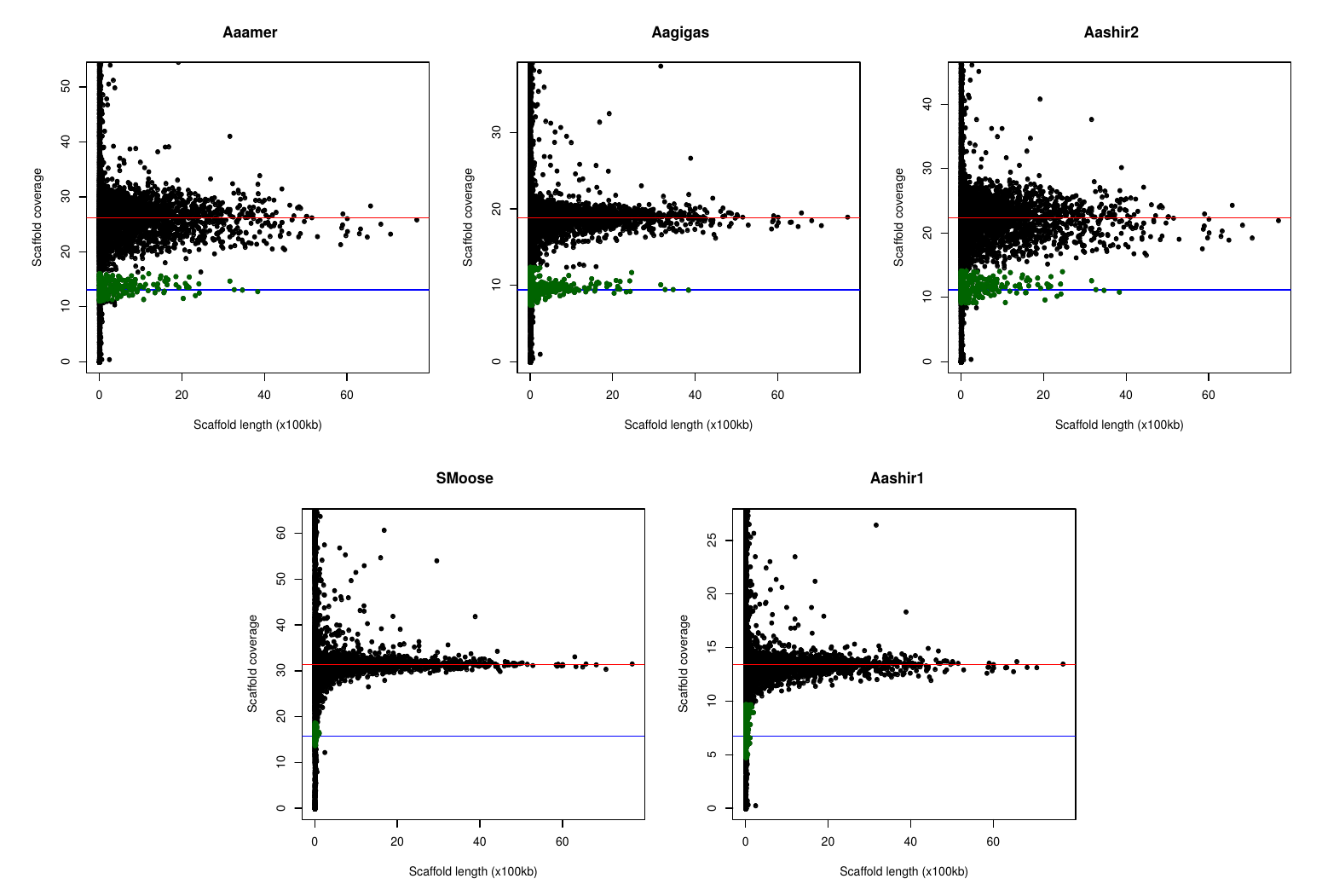


**Figure S1. Identification of scaffolds linked to chromosome X using sequencing coverage and scaffold length**. Green dots correspond to scaffolds assigned as linked to chromosome X. The red lines indicate the median coverage for all scaffolds. The blue lines represent half the median coverage which corresponds to the coverage of scaffolds linked to chromosome X.

**
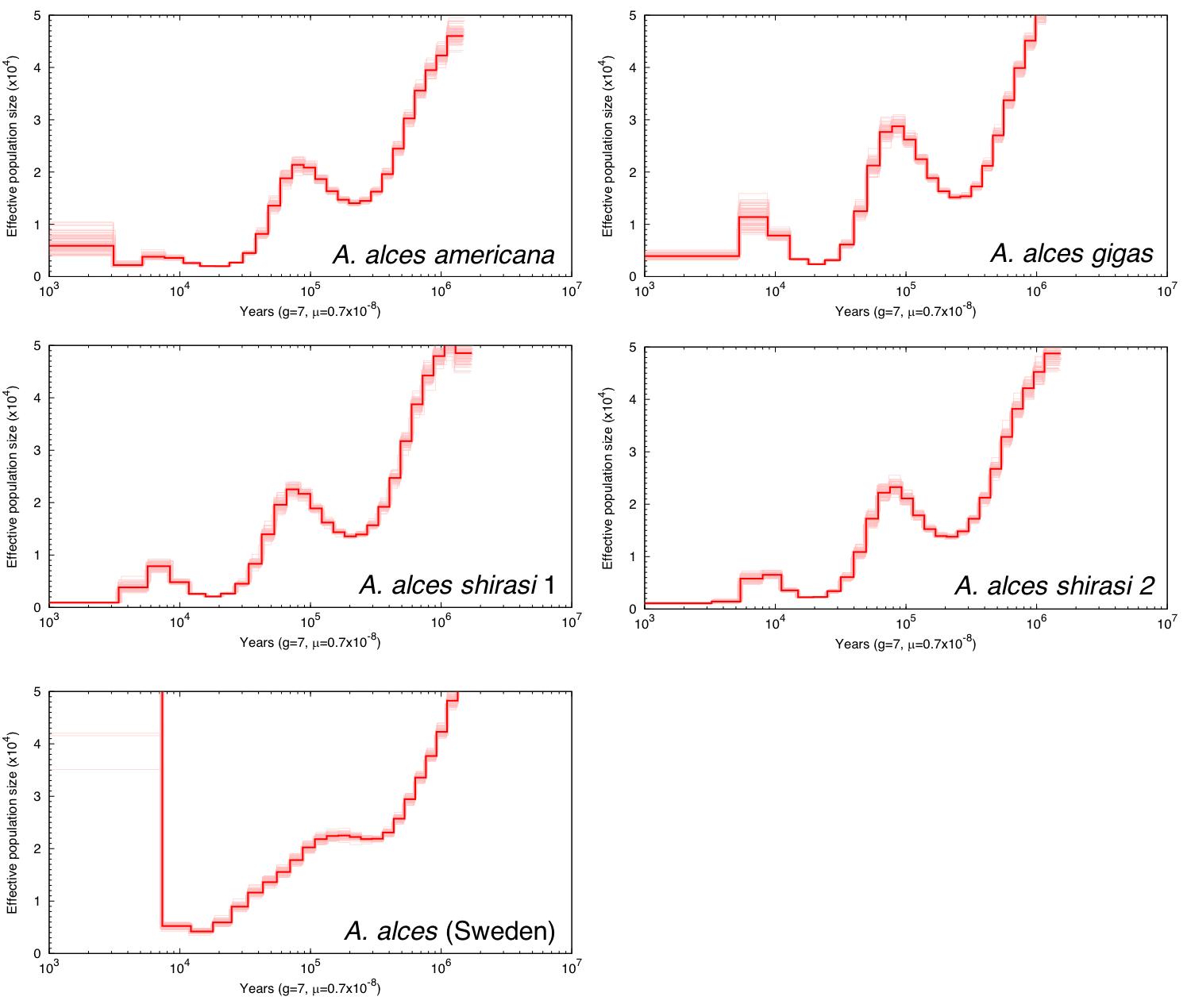
**

**Figure S2**. **Past demography for moose (*Alces alces*) using PSMC**. Thin lines represent 100 bootstrap runs. The x-axis corresponds to time before present in years on a log scale, assuming an estimated substitution rate of 0.7×10^−8^ substitutions/site/generation [[34]](https://paperpile.com/c/sGuQuK/c2NHQ) and a generation time of 7 years [[92]](https://paperpile.com/c/sGuQuK/6Qqgg). The y-axis corresponds to the effective population size *N*_e_.


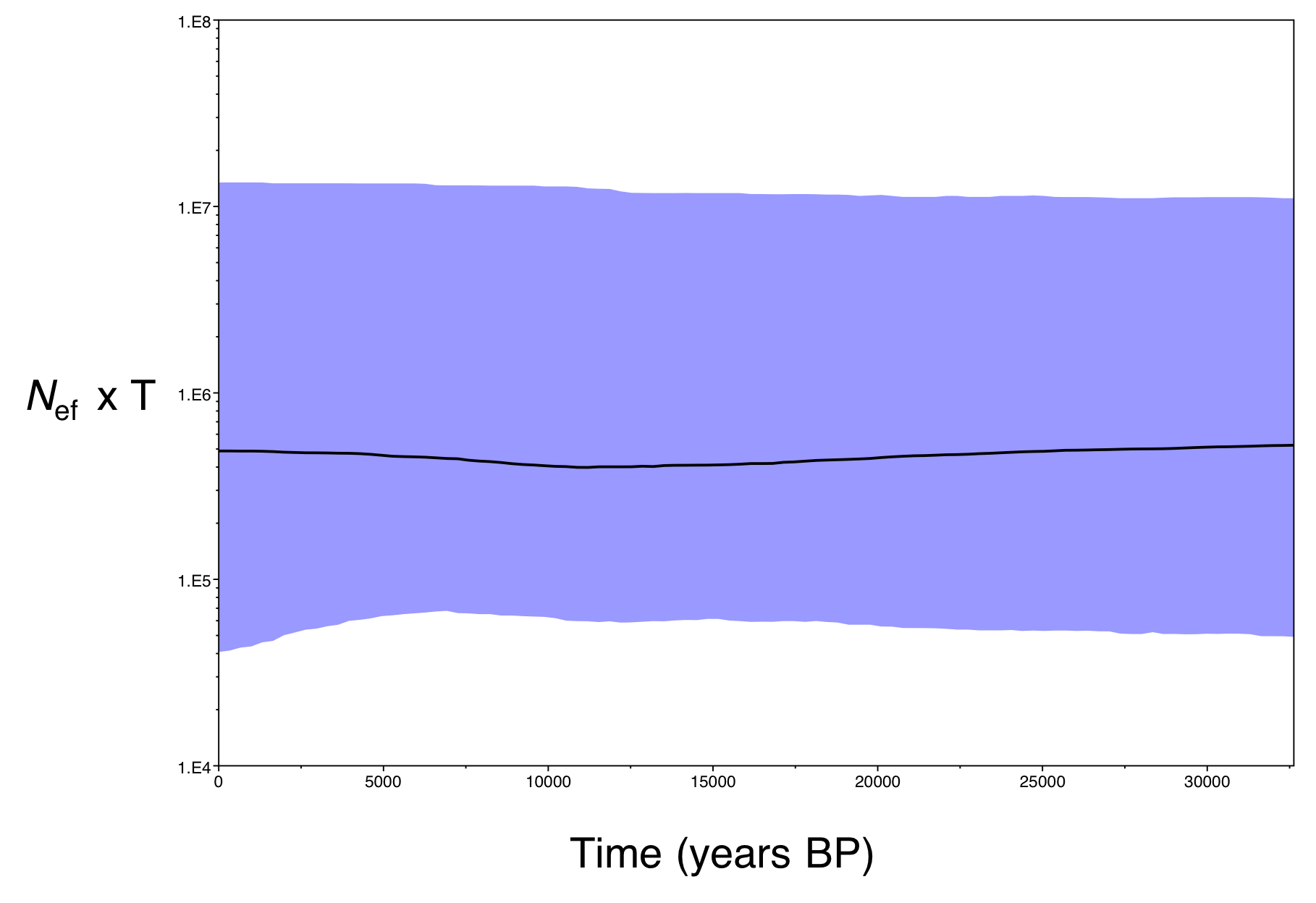


**Figure S3. Past demography for moose (*Alces alces*) using a Bayesian Skyline Plot** **(BSP).** Demographic reconstruction was inferred in BEAST using 14 European 16,693 bp mitogenomes. Timing of events was estimated assuming a mean rate of 9 x 10^-9^ substitutions/site/year based on Zurano et al. [[89]](https://paperpile.com/c/sGuQuK/7lqjd/?noauthor=1) and a standard deviation of 0.01. The x axis is in calendar years before present and y axis equals changes in effective population size (shown as the product of *N*_ef_ and generation time T). The black line is the median estimate and the blue lines show the 95% highest posterior density intervals.

**
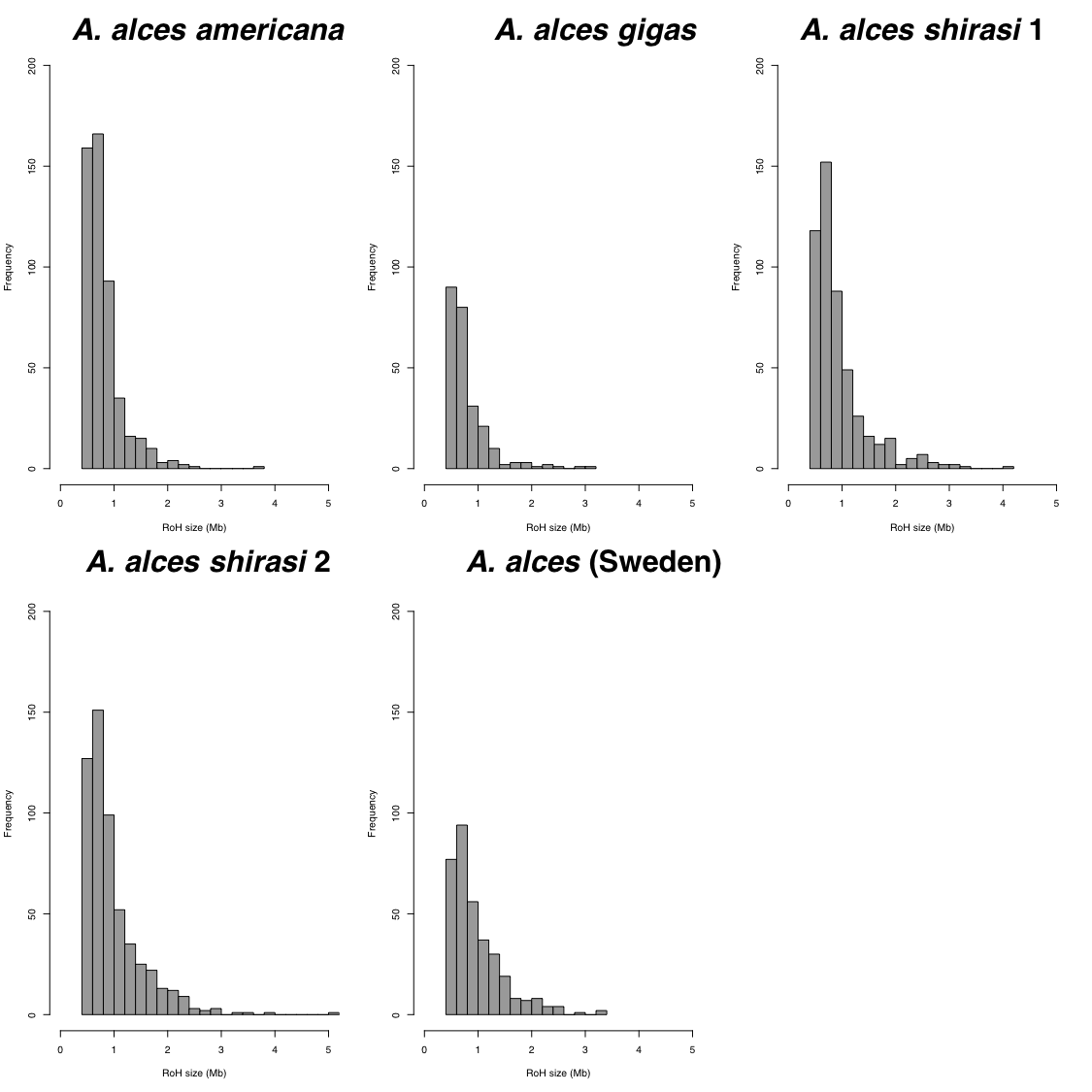
Figure S4**. **Distribution of runs of homozygosity (ROH) in moose (*Alces alces*).** ROH ≥500 kb are shown.
